# Benefits of a Switch from Intermittently Scanned Continuous Glucose Monitoring (isCGM) to Real-Time (rt) CGM in Diabetes Type 1 Suboptimal Controlled Patients in Real-Life: A One-Year Prospective Study ^§^

**DOI:** 10.3390/s21186131

**Published:** 2021-09-13

**Authors:** Yannis Préau, Sébastien Galie, Pauline Schaepelynck, Martine Armand, Denis Raccah

**Affiliations:** 1 Department of Endocrinology, Nutrition and Metabolic Diseases, University Hospital Sainte Marguerite, APHM, F-13385 Marseille, France; sebastien.galie@ap-hm.fr (S.G.); pauline.schaepelynck@ap-hm.fr (P.S.); denis.raccah@ap-hm.fr (D.R.); 2 Aix Marseille Univ, CNRS, CRMBM, F-13385 Marseille, France; martine.armand@univ-amu.fr

**Keywords:** real-time continuous glucose monitoring, intermittently scanned continuous glucose monitoring, type 1 diabetes, glucose biosensors, hypoglycemia, insulin resistance

## Abstract

The switch from intermittently scanned continuous glucose monitoring (isCGM) to real-time (rt) CGM could improve glycemic management in suboptimal controlled type 1 diabetes patients, but long-term study is lacking. We evaluated retrospectively the ambulatory glucose profile (AGP) in such patients after switching from Free Style Libre 1 (FSL1) to Dexcom G4 (DG4) biosensors over 1 year. Patients (*n* = 21, 43 ± 15 years, BMI 25 ± 5, HbA1c 8.1 ± 1.0%) had severe hypoglycemia and/or HbA1c ≥ 8%. AGP metrics (time-in-range (TIR) 70–180 mg/dL, time-below-range (TBR) <70 mg/dL or <54 mg/dL, glucose coefficient of variation (%CV), time-above-range (TAR) >180 mg/dL or >250 mg/dL, glucose management indicator (GMI), average glucose) were collected the last 3 months of FSL1 use (M0) and of DG4 for 3, 6 (M6) and 12 (M12) months of use. Values were means ± standard deviation or medians [Q1;Q3]. At M12 versus M0, the higher TIR (50 ± 17 vs. 45 ± 16, *p* = 0.036), and lower TBR < 70 mg/dL (2.5 [1.6;5.5] vs. 7.0 [4.5;12.5], *p* = 0.0007), TBR < 54 mg/dL (0.7 [0.4;0.8] vs. 2.3 [0.8;7.0], *p* = 0.007) and %CV (39 ± 5 vs. 45 ± 8, *p* = 0.0009), evidenced a long-term effectiveness of the switch. Compared to M6, TBR < 70 mg/dL decreased, %CV remained stable, while the improvement on hyperglycemia exposure decreased (higher GMI, TAR and average glucose). This switch was a relevant therapeutic option, though a loss of benefit on hyperglycemia stressed the need for optimized management of threshold alarms. Nevertheless, few patients attained the recommended values for AGP metrics, and the reasons why some patients are “responders” vs. “non-responders” warrant to be investigated.

## 1. Introduction

Continuous glucose monitoring (CGM) is the standard practice for improving the overall glycemic control providing different glucose metrics (time-in-range 70–180 mg/dL (TIR), time-below-range <70 mg/dL (TBR) and TBR < 54 mg/dL, coefficient of variation (%CV) of glucose, glucose management indicator (GMI), average interstitial glucose (IG) concentration), ameliorating sometimes HbA1c, the quality of life of type 1 diabetes (T1D) patients, and even hypoglycemia awareness in patients with hypoglycemic issue [1,2,3]. The choice of the best suited CGM devices for each patient is a major therapeutic decision, which can positively influence the risk of complications, acute or even chronic [4,5,6,7]. Two main systems are currently used in clinical practices, the intermittently scanned (is) CGM, also known as flash-monitoring, and the real-time (rt) CGM, both involving wearable electrochemical glucose biosensors with minimally-invasive implantation site. The most used biosensor for isCGM is the FreeStyle Libre version 1 (FSL1) [8,9,10,11,12] while several biosensors are used for rtCGM (e.g., Dexcom Platinum G4, G5 and G6; Medtronic Enlite, iPro or Guardian sensor) [8,9,10,11,12,13]. Each of the systems has specific differences. The isCGM system gives a value for IG concentration at 15 min intervals, requires scanning near the sensor placed on the skin at least every 8 h in order not to lose the glucose data recorded, does not require capillary glucose calibration, is devoid of alarms for hypo- or hyperglycemia excursions (for FSL1), and the sensor lifetime is 14 days [13,14,15]. The rtCGM system provides real-time access to IG concentration, with an IG concentration value delivered at 3 to 5 min intervals, with no need for scans, with trend curves permanently displayed on a monitor, requires twice-daily capillary blood glucose calibrations depending on the system (except for Dexcom G6 which is calibration-free), can be set to hypoglycemia/hyperglycemia threshold alarms [14,15], and the sensor lifetime is about 6 to 10 days [13]. It can be coupled to an insulin pump allowing insulin therapy management in a closed loop (automatic delivery system or hybrid closed-loop) [16]. Both systems are compatible with telemedicine support via dedicated platforms [17]. The accuracy and precision of the IG values reported by these devices is mainly evaluated by the mean absolute relative difference (MARD), i.e., the mean difference between interstitial and blood glucose values, most often over 24 h. It is well accepted that this MARD is variable from one sensor to another, and for the same sensor according to the range of glucose and other factors of variability (calibration, rate of change in glucose, several drugs, site of sensor installation, remaining sensor life) [15,18].

Recently, international guidelines have recommended the use of rtCGM devices compared to isCGM for patients with hypoglycemic issue (justified by threshold alarms) and/or treated by continuous subcutaneous insulin infusion (CSII) (justified by the possibility of coupling sensor and pump), this more in connection with sensor specific characteristics than based on comparative clinical data [19,20]. The few available comparative data on sensor types in patients naïve of CGM, via the I HART and the CORRIDA studies, showed a superiority of rtCGM device on TBR and on TIR compared to isCGM in hypoglycemia-prone T1D patients or not, followed for periods of 8 to 16 weeks [8,9,10]. Very recently, the ALERTT1 study reported a superiority of rtCGM vs. isCGM for TIR 70–180 mg/dL, HbA1c, TBR < 54 mg/dL and hypoglycemia awareness in 254 patients used to CGM (randomized in two groups using each a different sensor) followed up over 6 months [12]. For a patient already using CGM device but considered in treatment failure based on elevated HbA1c and/or persistent hypoglycemia issue, studies focussing on the effectiveness of a device switch seem more appropriate and therefore required [9]. In that purpose, we recently conducted and published an observational study in 18 T1D adult patients with suboptimal glycemic control (hypoglycemia issue and/or elevated HbA1c) despite using an isCGM sensor (FSL1, Abbott Diabetes Care Inc., Alameda, CA, USA), and taking advantage switching to rtCGM sensor (Dexcom G4 platinum or DG4, Dexcom Inc., San Diego, CA, USA) over a follow-up of 6 months [11]. We observed a clinically relevant increase in TIR (+9.5 percentage points or pp; *p* = 0.0015), a decrease in TBR < 70 mg/dL (−4.8 pp; *p* = 0.0044) and in the %CV (−4.6 pp; *p* = 0.0002), and no change in time-above-range (TAR). However, benefits plateaued between 3 and 6 months of DG4 use [11] raising the question about long-term improvements.

We thus studied retrospectively, in a real-life context, the glucose outcomes of switching from FSL1 to DG4 over a period of one year in a cohort of 21 T1D adult patients with suboptimal glycemic control, treated with CSII or multi-daily injections therapy (MDI).

## 2. Materials and Methods

### 2.1. Ethics

We performed a single-centre retrospective study of clinical practices in T1D patients followed-up in the Nutrition, Endocrinology and Metabolic Diseases department at the University Hospital Sainte Marguerite/AP-HM of Marseille (France). The study was approved and registered by the AP-HM local ethical board (AP-HM Health Data Portal No. 2019-173) and conducted in accordance with the Declaration of Helsinki. All participants gave written consent for their personal data collection for scientific research purpose.

### 2.2. Study Genesis and Objective

In routine clinical practice in our department, we face a moderate number of T1D patients on FSL1 sensor with suboptimal glycemic control (elevated HbA1c (≥8%) and/or severe hypoglycemia episodes) despite gold standard intensive insulin therapy and isCGM (FSL1 is a reimbursed sensor still mostly used in France). For these patients the change of insulin therapy management could be replaced by switching to rtCGM. Such patients, when treated with CSII, are therefore eligible for a predictive low-glucose suspend system. For reasons of personal comfort, many patients prefer using their same insulin pump, primarily a tubeless pump, or continuing MDI. When a switch to rtCGM was offered to patients, the only possibility was to prescribe DG4, the sole rtCGM sensor approved for reimbursement by the French health insurance system at the time of this study, outside sensor-augmented pump systems.

In the light of our previous study [11], our objective was to assess the potential persistent long-term benefit of switching from FSL1 to DG4 on glycemic control using metrics from the Ambulatory Glucose Parameters (AGP) report. The main evaluation criteria selected were TIR 70–180 mg/dL, TBR < 70 mg/dL, TBR < 54 mg/dL, TAR > 180 mg/dL, TAR > 250 mg/dL, average IG concentration, glycemic variability as %CV, GMI, the CGM system utilization rate and biological HbA1c.

### 2.3. Study Design and Patients

The electronic medical files of T1D patients (≥18 years) FSL1 users for at least 1 year and who started to switch to DG4 between December 2018 to October 2019 were selected. Patients were proposed changing FSL1 sensor when having severe hypoglycemia events (more than one episode of unconsciousness with glucose level <0.54 mg/dL during the last 12 months with FSL) or/and if elevated HbA1c level (≥8%). Both glucose sensors were used in compliance with manufacturers licenses. The medical files were excluded if patients regularly took paracetamol, had diabetic ketoacidosis during the last 3 months, were affected by a chronic progressive disease influencing diabetes (such as cancer, AIDS, viral hepatitis), received corticosteroid therapy for any disorders, were pregnant or planning pregnancy or breast-feeding, or had severe visual or hearing impairment, or reduced manual dexterity.

**Figure 1:**
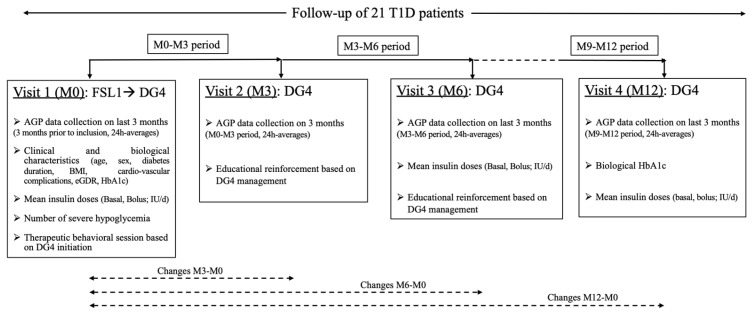
Study design. *Abbreviations*: AGP, Ambulatory Glucose Profile; BMI, body mass index; eGDR, estimated glucose disposal rate; FSL1, FreeStyle Libre version 1; DG4, Dexcom G4 platinum; HbA1c, plasma glycated hemoglobin A1c; T1D, type 1 diabetes.

Patients taking advantage of switching to DG4 participated in a therapeutic patient educational (TPE) program conducted by trained diabetologists and nurses, to promote adherence to the new sensor. Details regarding the TPE program are given in Figure 1 (study design) and in our previous study [11].

### 2.4. Data Collection

Patient characteristics, such as sex, age, diabetes duration, BMI, complications (retinopathy, nephropathy, coronary artery disease, carotid macroangiopathy and hypertension status), number of hypoglycemic episodes during the last year, were extracted from the medical electronic files. AGP data were downloaded from the Freestyle Libre Libreview (for FSL1) and the Dexcom Clarity platforms (for DG4). All glucose data given each 15 min from FSL1, and each 5 min from DG4 were used based on a 3-month data collection for FSL1 (last 3 months of use of this sensor for M0) and for DG4 (first 3 months of use for M3, M0-M3 period; last 3 months of use over 6 months for M6, M3-M6 period; and last 3 months of use over 12 months for M12, M9-M12 period). From all these glucose measurements, several glucose metrics were calculated (TIR, TBR, TAR, GMI, %CV, average glucose concentration) and reported on a 24-h basis average. Changes in glucose metrics were calculated by subtracting the averaged values obtained at M0 from the averaged values obtained at M3, M6 or M12 (values at M3 or M6 or M12–values at M0).

Data on biological HbA1c and mean insulin doses (basal, bolus) were collected retrospectively from the patient electronic files at M0, M6 (for mean insulin doses only) and M12.

We determined the insulin resistance score of the patients at M0 by calculating the estimated glucose disposal rate (eGDR mg/kg/min) [21] with the following equation [22]: eGDR_BMI_ = 19.02 − (0.22 × BMI, kg/m^2^) − (3.26 × hypertension status, [defined as 0 = no, 1 = yes]) − (0.61 × HbA1c, %). The status of hypertension was defined by the actual blood pressures (≥140/90 mmHg) or current use of any anti-hypertensive agents. The higher the score the lower the resistance to insulin.

### 2.5. Statistical Analysis

A power calculation was not possible since this study was retrospective. Data analysis was performed using GraphPad Prism version 9 (GraphPad Software, San Diego, CA, USA). Descriptive statistics (mean, standard deviation (SD), median, and 25th percentile or first quartile Q1, and 75th percentile or third quartile Q3) were performed and variable distributions were evaluated using the Shapiro-Wilk normality test. Data are thus presented as means (SD) when reaching normality or otherwise as medians [Q1;Q3]. When values followed a Gaussian distribution the paired t-test two-tailed was applied, otherwise the non-parametric Wilcoxon signed-rank test two-tailed for matched-pairs was used, when comparing data from FSL1 to DG4 (M3, M6 or M12), or from DG4 M3 to DG4 M6 or DG4 M12, or from DG4 M6 to DG4 M12. Association between patient characteristics at baseline (age, duration of diabetes, BMI, eGDR) and the changes of AGP metrics, or within glucose metrics, was tested using Spearman’s correlation-coefficient analysis. *p*-value < 0.05 was considered statistically significant.

## 3. Results

### 3.1. Patient Characteristics

The characteristics before the switch to DG4 of the 21 patients whom folder met the selection criteria are summarized in Table 1. Among them, 13 (62%) had elevated HbA1c (8.7 ± 0.7%), 8 (38%) had experienced at least one episode of severe hypoglycemia in the previous year, 3 (14%) had both criteria, and 10 (48%) had a high eGDR score (9.2 ± 0.9 vs. 5.6 ± 1.5 mg/kg/min) [21,22]. The average insulin doses calculated for the last 3 months were 0.26 ± 0.09 units/kg/d for basal dose and 0.54 ± 0.11 units/kg/d for total dose (basal dose + bolus dosages) with FSL1 (at M0), 0.26 ± 0.08 units/kg/d or 0.26 ± 0.09 units/kg/d for basal dose and 0.55 ± 0.13 units/kg/d or 0.54 ± 0.14 units/kg/d for total dose with DG4, at M6 or M12, respectively.

During the last 3 months of FSL1 use, the mean ± SD average number of scans within 24-hrs was 6.0 ± 4.1 (range: 1–15). The means ± SD of average threshold hypoglycemic or hyperglycemic alarms were, respectively, 71 ± 5 mg/dL or 239 ± 23 mg/dL when using DG4 sensor. Based on a 3-month average, the 24-h rates of sensor use for FSL1 or DG4 at M3, M6 and M12 were not significantly different (Table 2).

Regarding individual data, 52%, 71%, 57% and 67% patients showed a sensor use superior to 70% for FSL1 (range 78–100), for DG4 at M3 (range 77.5–100), at M6 (range 75.7–97.9) and at M12 (range 97.5–100), respectively. The recommendation of capillary blood glucose monitoring every 12 h for fingerstick calibration with DG4 was respected by all patients as verified at the time of trimestral visits all over the follow-up.

### 3.2. Impact of Switching from FSL1 to DG4 CGM Sensors on Glucose Metrics and HbA1c

The switch from FSL1 to DG4 at M3, M6 and M12 led to a higher TIR, and lower TBR < 70 mg/dL, TBR < 54 mg/dL, and %CV (Table 2). The TAR > 180 mg/dL was reduced only at 6 months of DG4 use, while the TAR > 250 mg/dL decreased earlier at 3 months as the average IG concentration and the GMI. Unexpectedly at 12 months, all TAR, average IG concentration and GMI returned to baseline values, i.e., close to FSL1 metrics. The biological HbA1c did not change after 12 months for the entire population studied (7.99 ± 1.04; mean change: −0.09 ±1.20 pp). But, considering apart the T1D patients with a baseline high level and who underwent a decrease (9 patients out of 13), the value decreased from 8.9 ± 0.8 to 7.7 ± 0.9 (mean change: −1.14 ± 0.85 pp; *p* = 0.0036; range: −0.4 to −2.8 pp).

Considering the period of DG4 use separately, while the decrease in %CV plateaued after 3 months up to 12 months, the TIR, all TAR, average IG concentration and GMI plateaued up to 6 months and then evolved significantly at 12 months by diminishing for TIR, or by re-augmenting for all TAR, average IG concentration and GMI. Al contrary, TBR < 70 mg/dL continued decreasing at 12 months and the decrease in TBR < 54 mg/dL plateaued at 6 months (decreasing trend at 12-month compared to 6-month, *p* = 0.055).

**Figure 2:**
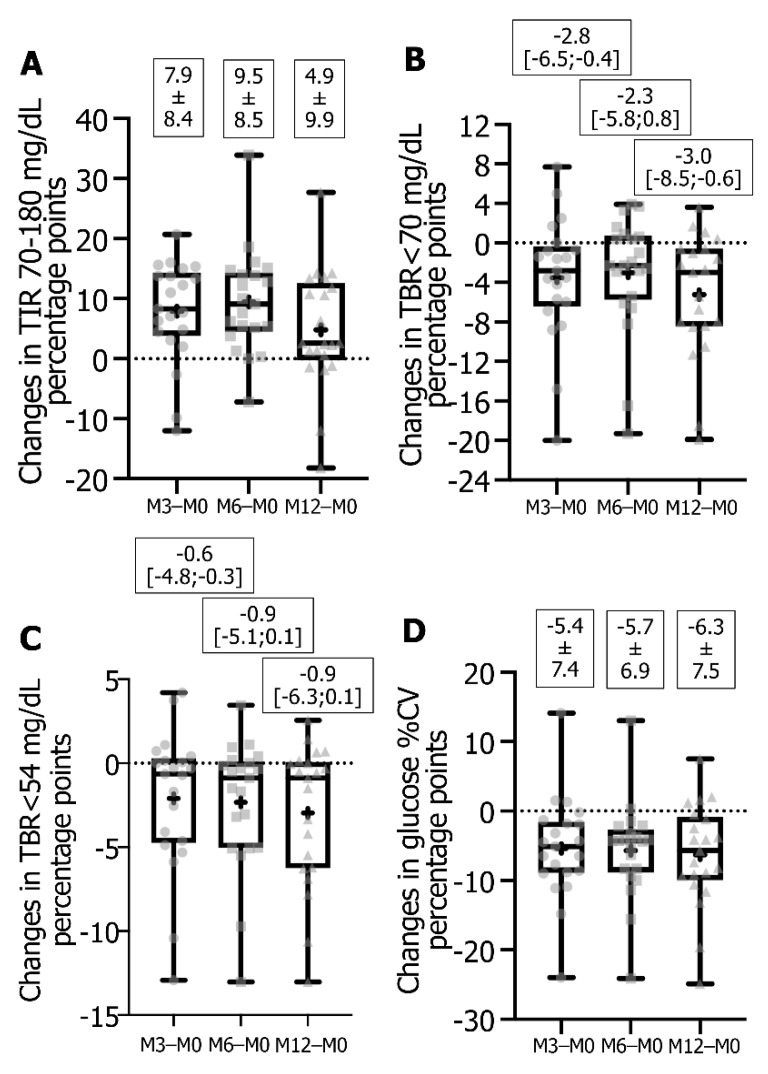
Box-and-whisker plot of changes in TIR (**A**), TBR < 70 mg/dL (**B**), TBR < 54 mg/dL (**C**) and glucose %CV (**D**). Data represented as plots are median, first quartile (Q1 or 25th percentile) and third quartile (Q3 or 75th percentile), min and max, mean (as a cross) and all individual points for changes calculated from DG4 parameter at M3 or M6 or M12 − FSL1 parameters expressed as percentage points. Values indicated are means ± SD or median [Q1;Q3]. Data are from 21 T1D followed-up patients. Abbreviations: %CV, glucose % coefficient of variability; DG4, Dexcom platinum G4; FSL1, FreeStyle Libre version 1; TBR, time-below-range; TIR, time-in-range.

Regarding the individual patient data from FSL1 to DG4 at M3 or M6, 18 or 19 patients out of 21 exhibited an increase in TIR (Figure 2A) with 12 or 11 reaching a change equal or superior to the mean value (i.e., +8.3 to +20.7 or +10.0 to +18.6 pp), 16 or 13 patients decreased TBR < 70 mg/dL (Figure 2B) with 11 reaching a change equal or superior to the median value (−2.8 to −20 or −2.3 to −19.3 pp), and 13 or 15 patients decreased TBR < 54 mg/dL (Figure 2C) with 11 having a change equal or superior to the median value (−0.6 to −10.4 or −0.9 to −13.0 pp). There were 18 or 19 patients who improved their glucose variability (Figure 2D) with 10 or 8 showing a decrease in glucose %CV equal or superior to the mean change value (−6.5 to −24.0 or −6.3 to −24.1 pp).

**Figure 3:**
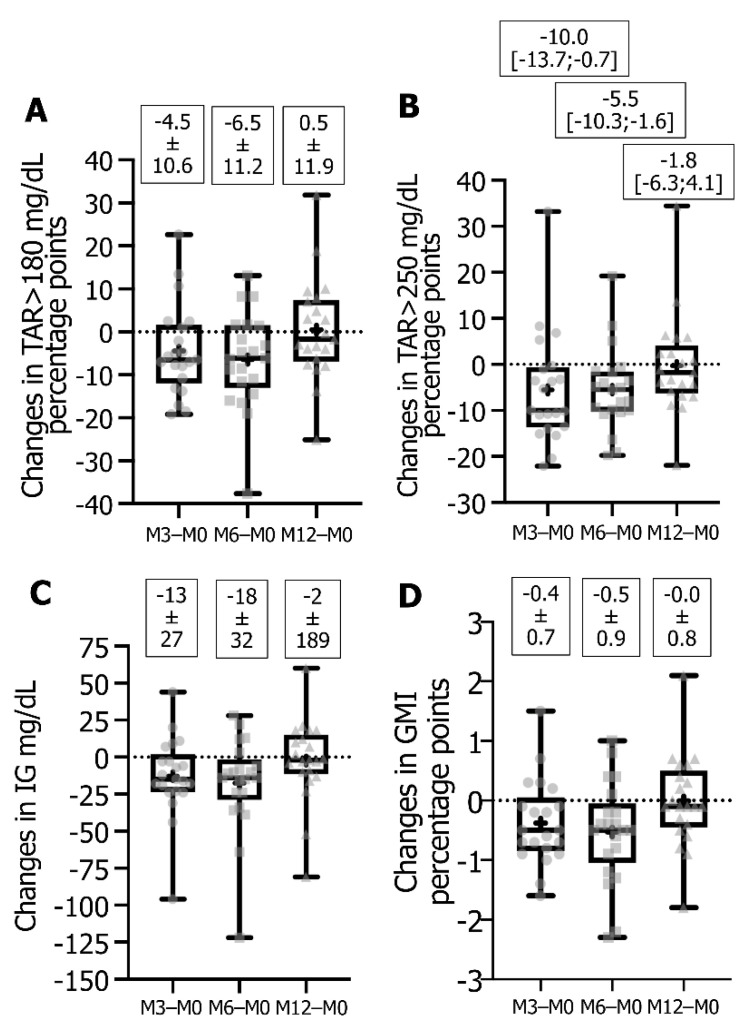
Box-and-whisker plot of changes in TAR > 180 mg/dL (**A**), TAR > 250 mg/dL (**B**), average IG concentration mg/dL (**C**) and GMI % (**D**). Data represented as plots are median, first quartile (Q1 or 25th percentile) and third quartile (Q3 or 75th percentile), min and max, mean (as a cross) and all individual points for changes calculated from DG4 parameter at M3 or M6 or M12 − FSL1 parameters expressed as percentage points except for average IG concentration which is in mg/dL. Values indicated are means ± SD or median [Q1;Q3]. Data are from 21 T1D followed-up patients. Abbreviations: DG4, Dexcom platinum G4; FSL1, FreeStyle Libre version 1; GMI, glucose management indicator; IG, interstitial glucose; TAR, time-above-range.

TAR > 180 mg/dL was diminished in 15 patients (Figure 3A), with 14 or 10 patients reaching a change equal or superior to the mean (−4.7 to −19.2 or −6.3 to −37.6 pp), and TAR > 250 mg/dL decreased in 17 or 18 patients (Figure 3B), with 11 patients exhibiting a change equal or superior to the median (−9.96 to −22.1 or −5.5 to −19.0 pp). The average IG concentration (Figure 3C) decreased in 16 patients, with 11 or 7 showing a change equal or superior to the mean (−15 to −96 or −24 to −122 mg/dL), and the GMI decreased in 16 patients (Figure 3D) with 12 or 11 reaching a change equal or superior to the mean (−0.5 to −1.4 or −0.5 to −2.3 pp).

For individual responses between FSL1 and DG4 at M12, 16 patients out of 21 increased TIR (Figure 2A) and 9 exhibited an increase equal or superior to the mean change value (+6.3 to +27.7 pp). For TBR < 70 mg/dL (Figure 2B), 17 patients showed a decrease with 11 of them reaching a value equal or superior to the median change value (−3.0 to −18.5 pp), and for TBR < 54 mg/dL (Figure 2C) a decrease was observed in 15 patients with 11 showing a change equal or superior to the median value (−0.9 to −13.0 pp). For glucose variability (Figure 2D), 17 patients improved %CV with 10 having a decrease equal or superior to the mean change value (−7.4 to −24.9 pp). For TAR > 180 mg/dL and TAR > 250 mg/dL (Figure 3A,B), 12 patients underwent a decrease with 11 reaching a diminution equal or superior to the mean change (−1.7 to −25.1 pp) or to the median change (−1.8 to −21.9 pp), respectively. Regarding average IG concentration (Figure 3C), 12 patients reached a decrease with 10 of them showing a change equal or superior to the mean (−3 to −81 mg/dL), and for GMI (Figure 3D) 12 patients exhibited a decrease all comprised between a change of −0.1 to −1.8 pp.

Simultaneously to a clinically relevant increase in TIR of at least +5 percentage points, after 6 or 12 months of DG4 use, 12 or 7 out of 21 patients improved TAR > 180 mg/dL, TAR > 250 mg/dL, GMI and average IG concentration concomitantly, while 10 or 9 improved simultaneously TBR < 70 mg/dL, TBR < 54 mg/dL, and %CV, and 6 of these patients improved all glucose metrics (data not shown).

To note, no severe hypoglycemia (event requiring the assistance from another person for administrating carbohydrates and/or glucagon, and/or brief hospitalization) was reported, nor ketoacidosis episode over the 12-month follow-up period.

### 3.3. Association between CGM Metrics

The associations between changes in CGM metrics (strong inverse association between TIR and TAR > 180 mg/dL and strong positive association between %CV and TBR < 70 mg/dL) after switching from FSL1 to DG4 at M6 were close to the ones previously published [11]. Additionally, herein, the change in TIR was inversely associated with the change in TAR > 250 mg/dL (r = −0.60, *p* = 0.004), and the change in %CV was positively associated with the change in TBR < 54 mg/dL (r = 0.63, *p* = 0.002).

**Figure 4:**
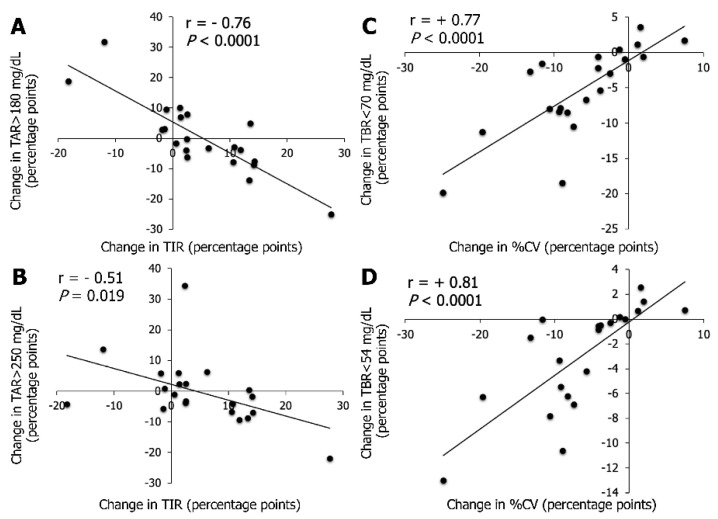
Linear association between changes obtained after switching from FSL1 to DG4 at M12 for TIR with TAR > 180 mg/dL (**A**) or with TAR > 250 mg/dL (**B**), and for glucose %CV with TBR < 70 mg/dL (**C**) or with TBR < 54 mg/dL (**D**). Changes were calculated from DG4 12-month parameters − FSL1 last 3-month parameters (values at M12 − values at M0) in 21 T1D patients and are expressed as percentage points. Correlation analysis was performed using Spearman’s rank correlation coefficient denoted by “r” for rho on the figure (*p* < 0.05). Equation of the line for A or B: change in TAR > 180 mg/dL= −1.02 × [change in TIR] + 5.43; change in TAR > 250 mg/dL = −0.52 × [change in TIR] + 2.20. Equations of the lines for C or D: change in TBR < 70 mg/dL = 0.65 × [change in %CV] − 1.16; change in TBR < 54 mg/dL = 0.43 × [change in %CV] − 0.24. Abbreviations: %CV, glucose coefficient of variability; DG4, Dexcom platinum G4; FSL1, FreeStyle Libre version 1; T1D, type 1 diabetes; TBR, time-below-range; TIR, time-in-range; TAR, time-above-range. No association was found between the number of scan/d with FSL1 at M0 and the evolution of metrics with DG4 over 12 months of follow-up.

The change in TIR after switching from FSL1 to DG4 at M12 was strongly inversely associated with the change in TAR > 180 mg/dL (Figure 4A), and moderately with the change in TAR > 250 mg/dL (Figure 4B). The change in TIR was moderately inversely associated with the change in GMI (r = −0.45, *p* = 0.04), and was not associated with the change in TBR < 70 mg/dL or TBR < 54 mg/dL. The change in %CV was only strongly positively associated with the change in TBR < 70 mg/dL (Figure 4C) and with the change in TBR < 54 mg/dL (Figure 4D).

### 3.4. Association between Patient Characteristics at Baseline and Changes in Metrics

Among the different characteristics of the patients at baseline, only the eGDR score showed an inverse association with the change in TAR > 180 mg/dL (r = −0.63; *p* = 0.0022), the change in TAR > 250 mg/dL (r = −0.49; *p* = 0.022), the change in average IG concentration (r = −0.64; *p* = 0.0017) and the change in GMI (r = −0.67; *p* = 0.0008) obtained at M12.

## 4. Discussion

Our real-life retrospective study reports an overall glucose profile improvement at one year of switching from FSL1 to DG4 in adults T1D patients with suboptimal glycemic control (elevated HbA1c and/or hypoglycemia issue). The main relevant clinical outcome was a significant average improvement of four targets of the glucose management [23] namely an increase in TIR (+4.9 pp), a decrease in %CV (−6.3 pp), as well as a decrease in TBR < 70 mg/dL (−3.0 pp) and in TBR < 54 mg/dL (−0.9 pp). It is important to emphasize that these two latter improvements agreed with the absence of severe hypoglycemia for the one-year period, and that the cited benefits occurred whatever the baseline glycemic problem and the insulin delivery modalities. However, on the average, while some improvements were accentuated (significant decrease for TBR < 70 mg/dL or decreasing trend for TBR < 54 mg/dL) or were persistent (%CV) on the long term by comparison to the mid-term follow-up, conversely, we observe a significant “re-ascent” of metrics associated with ambient hyperglycemia, namely a decrease in TIR (−4.61 pp), an increase in TAR > 180 mg/dL (+7.0 pp) and in TAR > 250 mg/dL (+5.20 pp), in GMI (+0.52 pp), and in average interstitial glucose concentration (+15 mg/dL).

As discussed in our previous mid-term switch-study [11], the explanations for glucose benefits reported over one year (TIR, TBR, %CV) is probably the consequence of switching to DG4 in combination with appropriate education follow-up (TPE program, the need for daily calibrations reinforcing the investment in the daily management of diabetes, threshold hypo/hyperglycemic alarms increasing the ability of patients to respond properly to glycemic excursions) [19]. More specifically, the prolonged or accentuated benefit, respectively, in %CV or TBR is corroborated by the existence of a positive strong association between these metrics as shown in our previous mid-term study [11], and confirmed herein, recalling that the intraday variability of glucose is largely influenced by exposure to hypoglycemia at different thresholds, and for any average interstitial glucose value [24]. To note that our TBR values were in the range of those obtained in T1D patients using Dexcom G4, G5 or G6 for at least 3 months, with a close hypoglycemia alarm threshold (<73 mg/dL) giving TBR < 70 mg/dL and TBR < 54 mg/dL around 4.7% and 1.5%, respectively [25]. Lower TBRs values could be possibly obtained using an alarm threshold ≥ 73 mg/dL, a value of 75 mg/dL being reported as optimal [25].

The reasons of the rise of all hyperglycemia metrics at 1 year follow-up warrant to be determined for solving this problem. Such observation should alert on the need for reinforced educational monitoring to ensure patient satisfaction with the device on a long-term, and to assess the proper management of alarms and to limit alarms exhaustion. Indeed, the threshold choices for audible alarms is clearly a central topic in the expected glycemic benefit with rtCGM system, the best threshold reported being 170 mg/dL for improving TAR [25]. Our hyperglycemia threshold alarm (239 ± 23 mg/dL) was probably too high for obtaining a relevant persistent change in hyperglycemia metrics on the long-term and should have be revised downwards over time in our patients. Since we confirm a negative association between TIR and TAR, and TIR and GMI, and none between TIR and TBR [11], it was not surprising that a re-ascension in hyperglycemia metrics was accompanied by a diminution in TIR, even if the final value remained significantly higher than with FSL1.

Concerning the evolution of HbA1c, we did not find any association with the evolution of TIR 70–180 mg/dL unlike randomized trials [26]. However, the strength of the association between TIR 70–180 mg/dL and HbA1c depends on the baseline HbA1c value, the duration of CGM data captured, the IG concentration average (stronger association if IG average is between 120 and 200 mg/dL) and factors affecting the accuracy of HbA1c assay [7,27].

Even though averages are needed in scientific publications, considering the benefits for each patient individually is more pertinent to a physician point of view. Thus, on the long term this therapeutical option led to the improvement of all hypoglycemic metrics plus %CV for 43% of the studied patients, and of all hyperglycemic metrics for 33% of them, concomitantly with a clinically relevant increase in TIR in both cases, with 29% patients improving all glucose metrics. The reasons why not all the studied patients underwent benefits deserve further interest. Another plausible explanation for such heterogeneity in improvement of glucose metrics might be the patients baseline characteristics and especially the insulin resistance level estimated by eGDR, as previously suggested [11] since this score was shown negatively associated with the change in hyperglycemia metrics. The clear impact of insulin resistance level and glucose management in T1D needs to be explored with a higher number of patients.

For discussing the achievement of the glucose target values according to the consensus recommendations from Advanced Technologies and Treatments for Diabetes (ATTD) [23], none average reached herein such thresholds whatever the CGM device used as in the other switch study [9], except for TBR < 70 mg/dL and TBR < 54 mg/dL after the use of DG4 for 12 months. If this achievement is discussed on an individual basis (Table 3), interestingly at 12 months of DG4 use after switching from FSL1, 2 to 3.5 folds more patients achieved %CV, hypoglycemia targets, TIR and the extreme hyperglycemic target, suggesting further some interest of such therapeutical option in patients with suboptimal diabetes control.

Limitations and strengths of our study must be addressed. One limitation was the small number of subjects due to the medical context to be studied meaning that our findings cannot be generalized but rather corresponded to personalized medicine. In addition, the heterogenous profile of our patients avoided subgroups analysis to better understand the factors explaining the extent difference of the benefits. Improvements on glycemic management by switching from a system to another one also depend on the accuracy of IG values, attested by the MARD, which is very variable between different sensors even from the same company [28,29,30,31,32], that was not further studied. No standardized questionnaires were retrieved in the medical folders for evaluating satisfaction and quality of life or the evolution of the feeling and fear of hypoglycemia. The strengths were performing a real-life protocol allowing a one-year follow-up of patients being their own control instead of studying two-arms of different patients using each a different sensor [8,10,12]. In addition, the small number of patients allowed an individual analysis by sessions of 3 months over 12 months providing a personalized vision of each patient evolution about CGM metrics. Moreover, real-life approach favoured the recruitment of patients with varied glycemic profiles and allowed to observe that the benefit of such CGMs’ switch, in our study context, could be suitable for any adult T1D patient responding to one or the other of the two main criteria of glycemic control, that are a sub-optimal HbA1c or hypoglycemia issue. Finally, the use of FSL1 and DG4 is still current in our clinical practices in France, as well as in other countries out of USA, underlining the usefulness of this type of study.

## 5. Conclusions

Our real-life over one-year study of a switch from isCGM (FreeStyle Libre version 1) to rtCGM (Dexcom G4), without changing the insulin therapy management, reports an overall glycemic benefit, despite a rise in hyperglycemia metrics comparatively to mid-time follow-up, highlighting the interest of such therapeutical option in specific patients having a suboptimal diabetes control. Future studies should focus on the reasons why some patients are “responders” and some “non-responders” to devices switch and explain the relative loss of long-term efficacy on the hyperglycemia metrics, especially in relationship with insulin resistance and via the management of alarms thresholds as much as possible it can be in a real-life context (alarms weariness, regular reassessment of thresholds, corrective behaviours if alarms are triggered). Furthermore, the real-time monitoring wearable biosensors are in significant progress since 2010 through rtCGM to help achieving a more regular glycemic balance over several years, which remains a crucial contribution for long-term health conditions in the context of a chronic disease like diabetes [13]. Thus, this technology of continuous interstitial glucose concentration monitoring in real time has led to the emergence of new closed-loop devices (or “artificial pancreas”) with the help of artificial intelligence, which are being evaluated on a long-term in real life in several ongoing studies [33,34]. In addition, soon, new glucose monitoring biosensors on atypical sites (eye, saliva, perspiration) possibly coupled with biosensing of other biomolecules associated with glucose metabolism (cortisol, lactate, ketone bodies) will open new perspectives in a more efficient individualized management of diabetic patients.

## Figures and Tables

**Table 1:** Baseline clinical characteristics of the patients included in the study.

Variables	T1D Patients (*n* = 21)
Female/Male, *n* (%)	13/8 (62/38)
Age, years (range)	43.2 ± 15.1 (21–75)
Duration of diabetes, years (range)	25.1 ± 13.6 (7–60)
BMI, kg/m^2^ (range)	25.3 ± 4.9 (18–39)
Complications	
Retinopathy, *n* (%)	8 (38)
Nephropathy, *n* (%)	4 (19)
Coronary artery disease, *n* (%)	1 (5)
Carotid macroangiopathy, *n* (%)	2 (9.5)
Hypertension, *n* (%)	8 (38)
HbA1c, % (range)	8.08 ± 1.04 (6.3–10.6)
eGDR, mg/kg/min (range)	7.28 ± 2.22 (1.7–10.4)
Severe hypoglycemic episode ^1^ last 12 months, *n* (%)	8 (38)
CSII, *n* (%)	18 (86)

Data are expressed as mean ± standard deviation (range) or number (percentage). ^1^ Defined as ≥1 symptom of hypoglycemia with unconsciousness, blood glucose level < 54 mg/dL. Abbreviations: BMI, body mass index; CSII, continuous subcutaneous insulin infusion; eGDR, estimated glucose disposal rate; HbA1c, plasma glycated hemoglobin A1c; T1D, type 1 diabetes.

**Table 2:** Impact of CGM switching from Freestyle Libre1 to Dexcom G4 platinum over 12 months on glucose metrics.

Variables	FSL1 M0	DG4 M3	DG4 M6	DG4 M12	P M3 vs. M0	P M6 vs. M0	P M12 vs. M0
GMI ^1^, %	7.98 ± 1.34	7.60 ± 1.08 *	7.45 ± 1.14 *	7.97 ± 1.15	0.0255	0.0099	NS
TIR 70–180 mg/dL ^1,2^, %	45.4 ± 16.0	53.3 ± 16.4 *	54.8 ± 16.0 *	50.2 ± 17.1	0.0003	<0.0001	0.0365
TBR < 70 mg/dL ^1,2^, %	7.0 [4.5;12.5]	4.6 [2.6;9.9] *	4.6 [4.6;8.8] *	2.5 [1.6;5.5]	0.0153	0.0450	0.0007
TBR < 54 mg/dL ^1,2^, %	2.3 [0.8;7.0]	1.3 [0.7;4.3] *	1.4 [0.5;2.7]	0.7 [0.4;0.8]	0.0441	0.0107	0.0073
TAR > 180 mg/dL ^1,2^, %	45.4 ± 19.3	41.0 ± 17.8 *	39.0 ± 18.0 *	45.9 ± 18.2	NS	0.0152	NS
TAR > 250 mg/dL ^1,2^, %	19.4 [9.3;32.2]	10.1 [5.8;21.5] *	10.1 [3.6;25.7] *	16.2 [8.7;30.5]	0.0127	0.0071	NS
Average IG ^1^, mg/dL	184.5 ± 46.2	171.7 ± 31.0 *	166.9 ± 32.7 *	182.0 ± 33.1	0.0433	0.0206	NS
CV ^1^, %	45.4 ± 8.3	40.0 ± 6.0	39.7 ± 6.3	39.1 ± 5.1	0.0032	0.0013	0.0009
Sensor use rate, %	78.0 [35.5;91.0]	90.7 [66.1;94.5]	82.0 [57.1;95.0]	84.6 [57.1;92.2]	NS	NS	NS

Data are expressed as mean ± standard deviation or median [Q1;Q3] from 21 T1D patients switching from FSL1 (M0) to DG4 followed-up at 3 months (M3), at 6 months (M6) and at 12 months (M12, endpoint). ^1^ Data represent average values calculated from the data collected on the ambulatory glucose profile report over 3 months (for M0 during the last 3 months of FSL1 use, for M3 during the first 3 months of DG4 use, and during the last 3 months of DG4 use for M6 and M12). ^2^ Data are expressed as percent of time over a 3 month-period. * Values significantly different compared to M12 considering only the period of DG4 use (paired student t-test two-tailed when Gaussian, or Wilcoxon matched-pairs signed rank test two-tailed when not gaussian; *p* < 0.05 significant; NS: not significant).Abbreviations: CGM, continuous glucose monitoring; %CV, glucose % coefficient of variation; DG4, Dexcom G4 platinum; FSL1, FreeStyle Libre version 1; GMI, glucose management indicator; HbA1c, plasma glycated hemoglobin A1c; IG, interstitial glucose; TAR, time-above-range; TBR, time-below-range; TIR, time-in-range.

**Table 3:** Number of patients achieving the targets recommended by the ATTD consensus for glucose metrics using FSL1 then switching to DG4.

Metrics	Target Values ^1^	FSL1 M0 ^2^	DG4 M6 ^2^	DG4 M12 ^2^
% CV, *n*	≤36%	2	7	6
TBR < 54 mg/dL, *n*	<1%	6	7	13
TBR < 70 mg/dL, *n*	<4%	4	7	14
TIR 70–180 mg/dL, *n*	>70%	1	5	3
TAR > 180 mg/dL, *n*	<25%	3	6	3
TAR > 250 mg/dL, *n*	<5%	2	7	4
GMI, *n*	<7%	6	8	4

^1^ Target values recommended by the ATTD 2019 [23]. ^2^ Data represent the *n* number out of 21 studied T1D patients reaching the targeted value for a given metric using FSL1 (M0) or after the switch to DG4 used over 6 (M6) or 12 (M12) months. Abbreviations: ATTD, Advanced Technologies & treatments for Diabetes); CGM, continuous glucose monitoring; %CV, glucose % coefficient of variation; DG4, Dexcom G4 platinum; FSL1, FreeStyle Libre version 1; GMI, glucose management indicator; T1D, type 1 diabetes; TAR, time-above-range; TBR, time-below-range; TIR, time-in-range.
